# Non-Hodgkins lymphoma of the nasal cavity: A case report

**DOI:** 10.1016/j.radcr.2023.08.073

**Published:** 2023-09-08

**Authors:** Konnor Kennedy, Cory Tremblay, Euan Zhang, Gordon Tsang, Ruba Kiwan

**Affiliations:** a NOSM University, Medical Sciences, 935 Ramsey Lake Road, Sudbury, ON P3E 2C6, Canada; b McMaster University Faculty of Health Sciences, Radiology, Hamilton, ON, Canada; c Health Sciences North, Sudbury, ON, Canada; d NOSM University, Medical Sciences, Sudbury, ON, Canada; e Northern Ontario School of Medicine—East Campus, Medical imaging, Sudbury, ON P3E 2C6, Canada; f Western University, Medical Imaging, London, ON N6A 3K7, Canada

**Keywords:** Lymphoma, Neuroradiology, Head and neck radiology, B-cell Lymphoma

## Abstract

We present a rare case of an 81-year-old woman presenting with acute left nasal blockage caused by a large nasal mass of unknown origin. The mass was subsequently diagnosed as diffuse large B-cell non-Hodgkin lymphoma (NHL). Nasal/paranasal space involvement in NHL is uncommon, representing only 0.2%-2% of cases. In this case, the nasal NHL mass exhibited a favorable prognosis, in contrast to previously reported sinonasal lymphomas with poor outcomes. The patient underwent excisional biopsy and was treated with 3 cycles of R-CHOP chemotherapy, resulting in complete resolution of the mass confirmed by a follow-up CT scan and no signs of disease after 1 year. Differentiating sinonasal lymphomas from other neoplasms can be challenging due to their variable morphology and location. Diffuse presentations of sinonasal lymphoma can aid in distinguishing them from discrete lesions associated with other sinonasal neoplasms. However, differentiation from acute invasive sinonasal infection remains difficult. MRI can help identify lymphomas through the characteristic hypointense T2 signal and diffusion restriction, with the combined use of CT to aid in differentiating masses of unknown morphology. Nonetheless, squamous cell carcinoma, which mimics lymphoma features on MRI, poses additional challenges to accurate identification. This case highlights the rarity of nasal NHLs, their potential for excellent prognosis, and the importance of diverse imaging techniques in their diagnosis and differentiation from other sinonasal pathologies.

## Introduction

Non-Hodgkin lymphoma (NHL) of the head and neck are relatively common, accounting for roughly 1/3 of all cases of extranodal disease. However, cases involving the nasal/paranasal space are much rarer, accounting for 0.2%-2% of the disease burden [1]. One particularly relevant characteristic of these malignancies is the predominance of nasal NHL's to be of NK/T-cell origin, with extra-nodal involvement of the tonsils, throat, and paranasal sinuses being predominantly B-cell lymphomas [2], [3], [4]. B-cell lymphoma typically has a much more positive prognosis. Sinonasal lymphoma generally have poor outcomes [2,5,6] because these masses are seldomly detected given their variable morphologic features and inconsistent location within the body. Sinonasal lymphoma can be discrete or infiltrative and vary in location, which makes them difficult to accurately identify and treat in a timely manner. We report a case of an 81-year-old woman presenting with acute left nasal blockage due to a mass of unknown origin.

This case report is significant for multiple reasons. The improbability of developing a new primary cancer in the nasal region is notable given the patient's history of resolved breast cancer and longstanding IgM monoclonal gammopathy of undetermined origin with limited bone marrow involvement. The new primary diffuse B-cell lymphoma mass developed in the nasal cavity and accounts for a very small proportion of nasal neoplasms. Nasal lymphomas are primarily T-cell in origin, whereas our patient's mass was of B-cell origin. The case report is also important, given the large differential list and difficulty with detection that is present with these nasal lesions, and awareness and vigilance is needed to ensure positive patient outcomes especially given the higher mortality rate if these lesions are not caught early.

## Case presentation

**Fig. 1:**
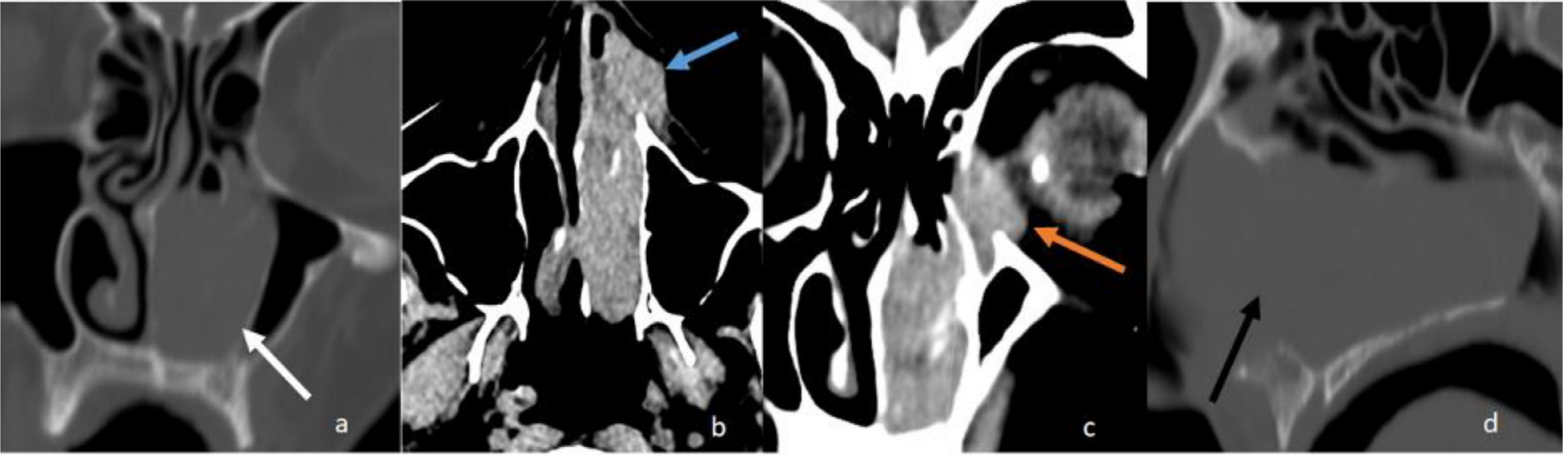
Paranasal sinus CT scan at presentation. (A) Coronal view bone window demonstrated mass centered in left nasal cavity with expansion of the nasal cavity and involvement of the inferior turbinate (arrow), (B) Axial view soft tissue window demonstrates extension into the nasal aperture, (C) Extension into the left medial orbit extraconal space, (D) Sagittal view bone window demonstrates nasal cavity expansion with no bone destruction. Differential diagnosis at this stage included: sinonasal carcinoma (including adenocarcinoma and squamous cell carcinoma), sinonasal granulomatosis with polyangiitis, and sinonasal melanoma.

**Fig. 2:**
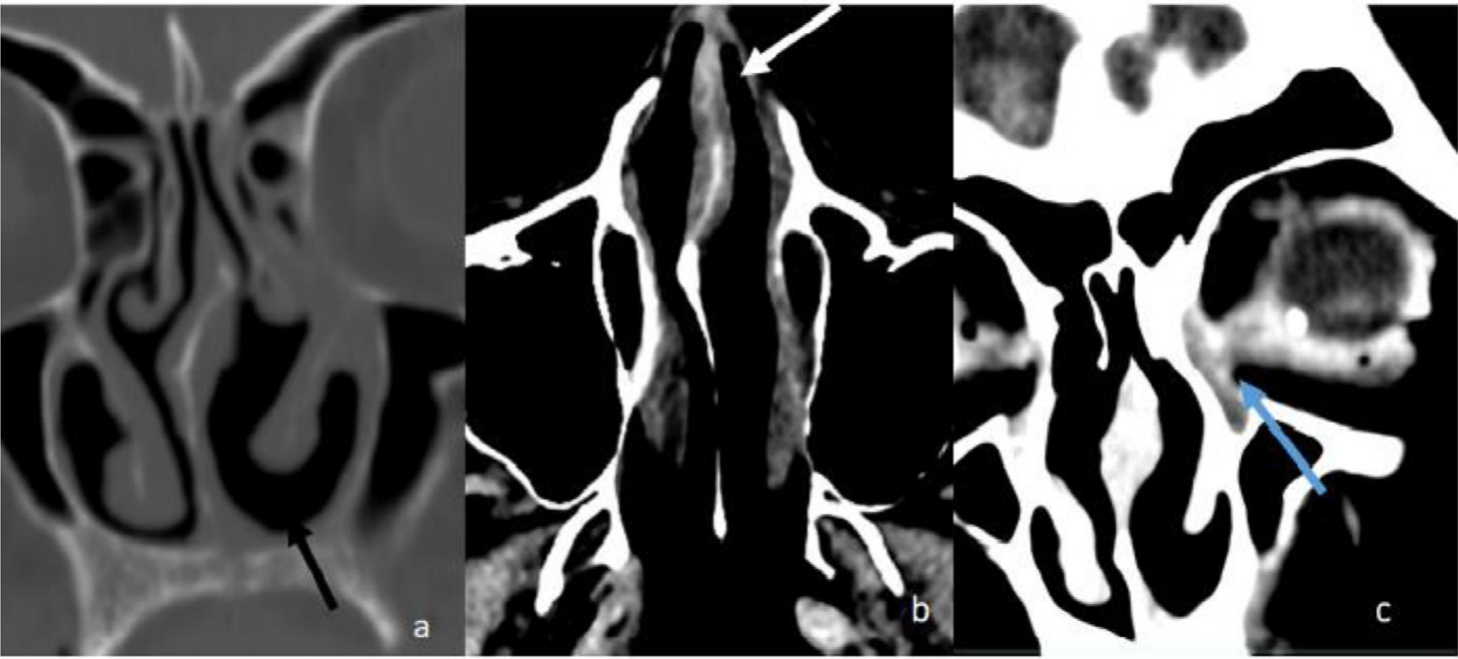
Follow up CT scan 3 months after treatment. (A) Coronal bone window at the level of the inferior turbinate demonstrates complete resolution of the left nasal cavity mass (B) Axial soft tissue window at the same level with patent left nasal cavity and (C) Coronal soft tissue demonstrates complete resolution of the left orbital component.

An 81-year-old woman presented with acute left nasal blockage to the local hospital. Physical examination was overall unremarkable except for a soft tissue mass affecting the left side of the face that was distorting the left nostril. There was also formation of nodular lesions along the left upper lip, below the nostril, and medial canthus of the left eye. There were no signs of cervical, supraclavicular, axillary, femoral or inguinal lymphadenopathy. No signs of hepatosplenomegaly. Computed tomography (CT) demonstrated a large 5.5 cm left nasal mass expanding the nasal cavity, extending to the left aperture and medial orbit (Fig. 1). Excisional biopsy under anterior rhinoscopy revealed a diffuse large B-cell NHL, confirmed by immunohistochemical staining. This revealed a mYc translocation, suggestive of aggressive B-cell lymphoma. BCL2 and BCL6 disruptions were not identified. Staging chest, abdomen and pelvis scans demonstrated no lymphadenopathy. She did not have an MRI scan. Her past medical history is significant for breast cancer, which was treated successfully and has been in remission for many years. She showed an asymptomatic elevation of serum IgM monoclonal gammopathy, but this has been stable for more than 10 years with no treatment and minimal bone marrow involvement. She was treated with 3 cycles of chemotherapy utilizing R-CHOP (rituximab, cyclophosphamide, hydroxydaunorubicin, vincristine, prednisone). A follow-up CT scan after 3 months demonstrated complete resolution of the mass (Fig. 2), with no clinical signs of disease 1 year later.

## Discussion

We present a rare case of nasal lymphoma that demonstrated excellent prognosis. Previously published case reports of sinonasal lymphoma had poor outcomes [2,5,6], seeing as these sinonasal masses are seldomly detected. Sinonasal lymphoma are often discrete or infiltrative, and variable in location. On CT, lymphomatous masses exhibit soft tissue density that is iso or hyperattenuating compared to muscle depending on the nucleocytoplasmic ratio. CT offers the advantage of assessing for bony remodeling, or bone destruction compared to MRI. On MRI, lymphomatous lesions tend to exhibit isointense signal on T1, and hypointense signal on T2-weighted images, as well as avid solid enhancement on post-gadolinium T1-weighted images, and restricted diffusion. However, diffuse lymphoma and acute invasive sinonasal infection can also appear similar. On MRI, hypointense signal on T2-weighted images and diffusion restriction of sinonasal lymphoma is quite characteristic compared to other neoplasms, which tend to exhibit T2 hyperintense signal and less diffusion restriction. Conversely, squamous cell carcinoma can be densely cellular and mimic features of lymphoma on MRI, which complicates accurate identification of these masses [7].

## Conclusion

Given the variable morphology and location of sinonasal lymphomas, distinguishing these neoplasms can be very challenging. In general, diffuse presentations of sinonasal lymphoma can be distinguished from other sinonasal neoplasms, which mostly present as discrete lesions. It was challenging confirming this nasal lymphoma diagnosis because they are rarely identified, have similar appearance to other neoplasms, and their unlikely tumor origin given the presenting location. Our case report adds important data to the literature of rare nasal lymphomas seeing as this particular lymphoma had such a positive response to treatment despite its rare presentation.
